# Editorial: Reward- and aversion-related processing in the brain: translational evidence for separate and shared circuits

**DOI:** 10.3389/fnsys.2015.00147

**Published:** 2015-10-29

**Authors:** Dave J. Hayes, Georg Northoff, Andrew J. Greenshaw

**Affiliations:** ^1^Brain, Imaging and Behaviour – Systems Neuroscience, Toronto Western Research Institute, Toronto Western Hospital, University Health Network, University of TorontoToronto, ON, Canada; ^2^Mind, Brain Imaging and Neuroethics Research Unit, Institute of Mental Health Research, University of OttawaOttawa, ON, Canada; ^3^Brain and Consciousness Research Center, Graduate Institute of Humanities in Medicine, Shuang Ho Hospital, Taipei Medical UniversityTaipei, Taiwan; ^4^Centre for Cognition and Brain Disorders, Hangzhou Normal UniversityHangzhou, China; ^5^Department of Psychiatry, University of AlbertaEdmonton, AB, Canada

**Keywords:** affective neuroscience, affective disorders, appetitive, aversive, reward, punishment, reinforcement, translational research

The dynamic evaluation of experience is existentially essential. Assigning value to events and objects drives neural development and plasticity and impacts our changing perceptual interpretations of the world and future behaviors (Nelson et al., 2014). The affective foundation of behavior provides more than a mere phenomenological “coloring” of experience. In fact, affect may be an inseparable component of sensation and cognition instead of an oft-considered byproduct (Inzlicht et al., 2015), and translational neuroanatomical evidence suggests that the major brain areas and tracts involved appear largely conserved across species (Panksepp, 2011).

Less clear are the neural underpinnings of valuative processing which give rise to positive and negative affective experience, appetitive/aversive encoding, reward/punishment-related reinforced behaviors, and feelings/emotions. There are many outstanding questions in this field—in addition to contention about precise definitions of terms such as emotion (Izard, 2009; Madan, 2013). Are appetitive and aversive stimuli encoded in similar brain areas? If so, do they share neural circuits and mechanisms? Do they function independently, in parallel, or is their cross-talk more complicated than this?

In this Research Topic, a number of authors have explored themes related to these fundamental questions at very different levels. Chris Madan uses a broad psychological-conceptual perspective with his presentation of the SIMON framework, which considers the interplay between the constructs of affect, reward, and motivation (Madan, 2013). This interplay could help contextualize findings showing that prior exposure to unpleasant images, inducing negative affect, can reduce reward-related responding in a reaction time task, even when motivation to perform is high. This framework also underscores how narrowly-focused experimental designs can advance our understanding of a given concept while also hindering a full appreciation of its real-world relevance.

Cross-conceptual thinking also helps elucidate the context-dependence of affective experience. Stimuli that follow painful events, and signal relief, can share neurophysiological characteristics with rewards but be reported as unpleasant. Andreatta et al. (2013) showed that the prediction of a painful stimulus differentially modulated a person's physiological output and behavioral reports. Both predictable and unpredictable conditioned stimuli following a painful shock acquired implicitly positive valences (i.e., skin responses consistent with relief), but only the predictable stimulus was reported as pleasant, while the unpredictable stimulus was said to be highly unpleasant. This speaks to the subjectivity and malleability of pain experience, and also to the context-dependence and interplay of affect, value and motivation alluded to by Madan.

Authors here have also explored biological mechanisms associated with valuative processing in simpler animals. Sinakevitch et al. (2013) looked at octopamine receptor, AmOA1, distribution in honey bee and fruit fly neurons, as octopamine-containing neurons (a homolog of dopamine) are involved in reinforcement and neural plasticity. In clever cross-species experiments, they revealed similar expression patterns and highlighted the importance of octopamine on the modulation of local GABAergic interneurons, which could help to clarify the mechanisms underlying food-odor reinforcement. Ducrot et al. (2013) underscored the involvement of glutamatergic AMPA and NMDA receptors in electrical brain self-stimulation reinforced behavior in rats. Blocking AMPA receptor function in the anterior ventral tegmental area decreased appetitive responding, perhaps related to reduced excitatory input to dopaminergic cells, while NMDA receptor blockade in more posterior areas increased appetitive responding, which likely reflects GABAergic disinhibition. Hayes' (2015) work echoes that of Sinakevitch and Ducrot by proposing that GABA-containing cells play a central role in valuative processing and suggests that long- and short-range GABAergic circuitry likely contributes to both the integration/cross-talk and differentiation of appetitive and aversive signals.

Human neuroimaging studies asked questions about emotional responsivity, behavioral control, and substance abuse at the whole-brain level. Lee et al. (2013) showed that a vasopressin V1a receptor antagonist could reverse the effects of vasopressin-induced amygdalar BOLD deactivations associated with the presentation of aversive faces. The antagonist also resulted in reduced activation to angry faces in the right temporoparietal junction, precuneus, putamen and medial prefrontal cortex. Nakao et al. (2013) used near-infrared spectroscopy to show that resting state signals in the dorsal portion of the medial prefrontal cortex (which contains the area identified by Lee et al. above) are negatively correlated with harm avoidance (a personality trait characterized by increases in aversive states such as worrying and pessimism), while novelty seeking (a trait reflecting exploratory behavior, impulsivity, and increased substance abuse risk) was reflected in a more ventral area. Brown et al.'s (2015a,b) behavioral-fMRI findings suggest that one must be careful not to conflate high-risk behavior with impulsivity, even if the two are behaviorally correlated, as brain responses associated with each do not largely overlap. Young adults who reported high-risk behaviors showed reduced activations during response inhibition in right orbitofrontal cortex and ventromedial prefrontal cortex, while impulsive people showed reduced activity in right posterior orbitofrontal, dorsomedial prefrontal, and perigenual anterior cingulate cortices. This group also showed increased lateral prefrontal cortex activation for aversive NoGo vs. aversive Go trials, irrespective of impulsivity or high-risk scores. Adolescents, however, showed very similar BOLD correlations with risk- and impulsivity-related scores although greater activation was noted in the lateral prefrontal cortices for neutral vs. aversive distractors—suggesting some differences in emotional-motor processing as a function of age.

Stewart et al. (2013) compared those with problem (*n* = 18) or stopped (*n* = 15) drug use and healthy controls in an fMRI study combining a two-choice affective prediction task with interoceptive challenges. Blunted frontocingulate activations during aversive interoceptive stimuli, and increased inferior frontal gyrus activation during punished feedback trials, in those with problem drug use may reflect goal-directed impairments in the face of negative external and internal challenges. Lominac et al. (2014) looked at stimulant-induced neural changes in biobehavioral experiments in mice. They showed that low subchronic doses of methamphetamine are capable of inducing changes within the mesocorticolimbic system, and that pre-existing differences in accumbens dopaminergic signaling—as seen in mice bred for high vs. low consumption—may predict a resistance to addiction.

Two final papers provide a more holistic view by reviewing the literature on valuative processing from a basic and psychiatric translational perspective. Bissonette et al. (2014) piece together the few animal and human studies employing both appetitive and aversive stimuli to help distinguish value- and salience-related neural mechanisms, finding a cross-species network of cortical (e.g., orbitofrontal to parietal) and subcortical (e.g., ventral tegmental area to substantia nigra pars compacta) regions which show preferences for each. Griffiths et al. (2014) used a similar translational approach, but instead considered that dysfunctional value-related decision making circuits may be the lynchpin to common psychiatric symptoms. They raise concerns about considering “decision-making” as a unified concept, and suggest that the strongest findings are from studies with translatable processes, such as those involving associative learning and goal-directed action tasks.

Advancements about “reward” and “fear” circuits, which have dominated the literature, have gradually paved the way for a more nuanced conceptualization of valuation in the brain. The mesocorticolimbic system can no longer be categorized as a “reward” or a “dopaminergic” circuit, nor can the amygdala be deemed the “fear center.” Importantly, identification of the interplay between positive and negative affective brain circuits, noted here and elsewhere (Vickery et al., 2011; Hayes et al., 2014; Lindquist et al., 2015), is highlighting the complexity of such networks. The primary goal of this Research Topic was to help identify some of the strengths of this approach and to help generate new hypotheses about how to better apprehend affective circuits.

## Conflict of interest statement

The authors declare that the research was conducted in the absence of any commercial or financial relationships that could be construed as a potential conflict of interest.
